# Insights into the skin microbiome dynamics of leprosy patients during multi-drug therapy and in healthy individuals from Brazil

**DOI:** 10.1038/s41598-018-27074-0

**Published:** 2018-06-08

**Authors:** Paulo E. S. Silva, Mariana P. Reis, Marcelo P. Ávila, Marcela F. Dias, Patrícia S. Costa, Maria L. S. Suhadolnik, Bárbara G. Kunzmann, Anderson O. Carmo, Evanguedes Kalapotakis, Edmar Chartone-Souza, Andréa M. A. Nascimento

**Affiliations:** Departamento de Biologia Geral, Instituto de Ciências Biológicas, Universidade Federal de Minas Gerais, Belo Horizonte, Minas Gerais Brazil

## Abstract

Leprosy is a chronic infectious peripheral neuropathy that is caused by *Mycobacterium leprae*, and the skin is one of its preferred target sites. However, the effects of this infection on the skin microbiome remain largely unexplored. Here, we characterize and compare the lesional and non-lesional skin microbiomes of leprosy patients and healthy individuals through the deep sequencing of 16 S rRNA genes. Additionally, a subset of patients was monitored throughout the multi-drug therapy to investigate its effect on the leprous skin microbiome. Firmicutes-associated OTUs (primarily *Staphylococcus*) prevailed in healthy individuals. By contrast, Firmicutes was underrepresented and Proteobacteria was enriched in the patients’ skin, although a single dominant taxon has not been observed at a finer taxonomic resolution. These differences can be explained by the significant decrease in *Staphylococcus* and *Streptococcus* as well as the enrichment in *Brevundimonas*. The overrepresentation of *Micrococcus* in patients is also remarkable. Genus-level compositional profiles revealed no significant intrapersonal difference between lesional and non-lesional sites. Treatment-associated changes indicated a loss of diversity and a shift in the community composition, with stronger impacts on the OTUs that are considered indigenous bacteria. Therefore, the molecular signatures associated with leprosy identified herein might be of importance for early diagnostics.

## Introduction

The indigenous skin microbiome is of great importance for human health, since it can promote resistance to pathogen colonization in addition to its essential role in physiology and immunity^1,2^. Over the last decade, the development of deep sequencing technologies has had a profound impact on the understanding of the human microbiome, providing important insights into the link between microbial ecology and host health^3^.

Several cutaneous disorders such as atopic dermatitis, psoriasis, and leprosy are associated with shifts in the microbiome^4,5^, although its contribution to the pathophysiology of these disorders is inconclusive^6^. In individuals with atopic dermatitis, Firmicutes are overrepresented, with a high prevalence of staphylococci, including both *Staphylococcus epidermidis* and *S. aureus*^7^, whereas the psoriatic microbiome is enriched with Firmicutes and Actinobacteria^8^. By contrast, the lesional leprous skin microbiome showed an overrepresentation of Proteobacteria and an underrepresentation of Actinobacteria^4^.

Leprosy, which is also known as Hansen’s disease, is a chronic infectious peripheral neuropathy caused by *Mycobacterium leprae* that can be successfully treated with multi-drug therapy (MDT). *M. leprae* preferentially targets the skin, the peripheral nerves, nasal mucosa, eyes, and reticulum-endothelial system^9^. The World Health Organization report on leprosy showed that over the last decade (2006–2015), the global caseload has fallen by almost 21%. In 2015, a total of 211,973 new cases were reported in 108 countries, from which only three accounted for most cases (60%, 13%, and 8% in India, Brazil, and Indonesia, respectively). Furthermore, although leprosy is no longer considered a public health problem worldwide, discrimination and stigmatization against affected people pose huge barriers to equitable treatment and social inclusion^10^.

Many investigations on the healthy skin microbiome have been performed in western countries, particularly in Americans^1,11–14^ and to a lesser extent in eastern countries^15–17^. These studies have revealed that skin microbiomes from western individuals are prominently populated by Actinobacteria and Firmicutes, followed by Proteobacteria and Bacteroidetes. In eastern countries, the same four bacterial phyla were reported, although at different relative frequencies.

From a wealth of data based on deep sequencing technologies, one of the most important conclusions is that biogeography and individuality shape the structural and functional composition of the human skin microbiome^18^. Additionally, many factors can promote changes in microbial communities, including intrinsic conditions such as genetic variations, the immune system, the occurrence of the disease and the ethnic group^19,20^, and extrinsic perturbations such as the environment, lifestyle, probiotics, diet or antimicrobial treatments^21,22^.

Given the highly personalized nature of the skin microbiome and the limited data on this subject^3^, we explored the general features of healthy and leprous skin in community and clinical settings. Moreover, a longitudinal study with a subset of patients under treatment was performed to investigate the impact of MDT on lesional and non-lesional leprous skin microbiomes.

## Results

### Sequencing overview

This is the first report on the composition and diversity of healthy and leprous skin microbiomes during MDT to be performed through the deep sequencing of 16 S rRNA genes in Brazil. A total of 10,403,990 high-quality reads from 140 samples (control n = 19, and for IVL, IVNL ML, MNL, LL, LNL, and PTL PTNL, see Table 1) were grouped into 20,315 OTUs. The numbers of reads and taxonomic assignments for these OTUs are presented in Supplementary Dataset 1.

**Table 1:** Sample collection map of leprosy patients included in this study.

*Patients	IV	M	L	PT
1	0	6		
2	0	6	12	
3	0			
4	0	5	11	3
5	0	7	11	
6	0	7		2
7	0		10	1
8	0	9		
9	0	8		4
10	0			
11	0			
12		9		5
13	0	8	12	5
14	0	6		
15	0	5	12	4
16	0	6	11	4
17	0		11	5
18	0	7		
19	0			
20	0			
21	0			
22	0	6	12	5
23	0			
24	0			
25	0			
26	0			
27	0	5		

The patients are identified by numbers (1 to 27). The number inside the column indicates the sample month during the study.

IV, initial visit; M, middle-treatment stage (between the 5^th^ and 9^th^ months of multi-drug therapy - MDT); L, late-treatment stage (between the 10^th^ and 12^th^ months of MDT) and PT, post-treatment stage (between the 1^st^ and 5^th^ months following the end of MDT).

^*^For each patient, samples were taken from non-lesional and lesional sites.

### Alpha and beta diversity analyses

**Figure 1:**
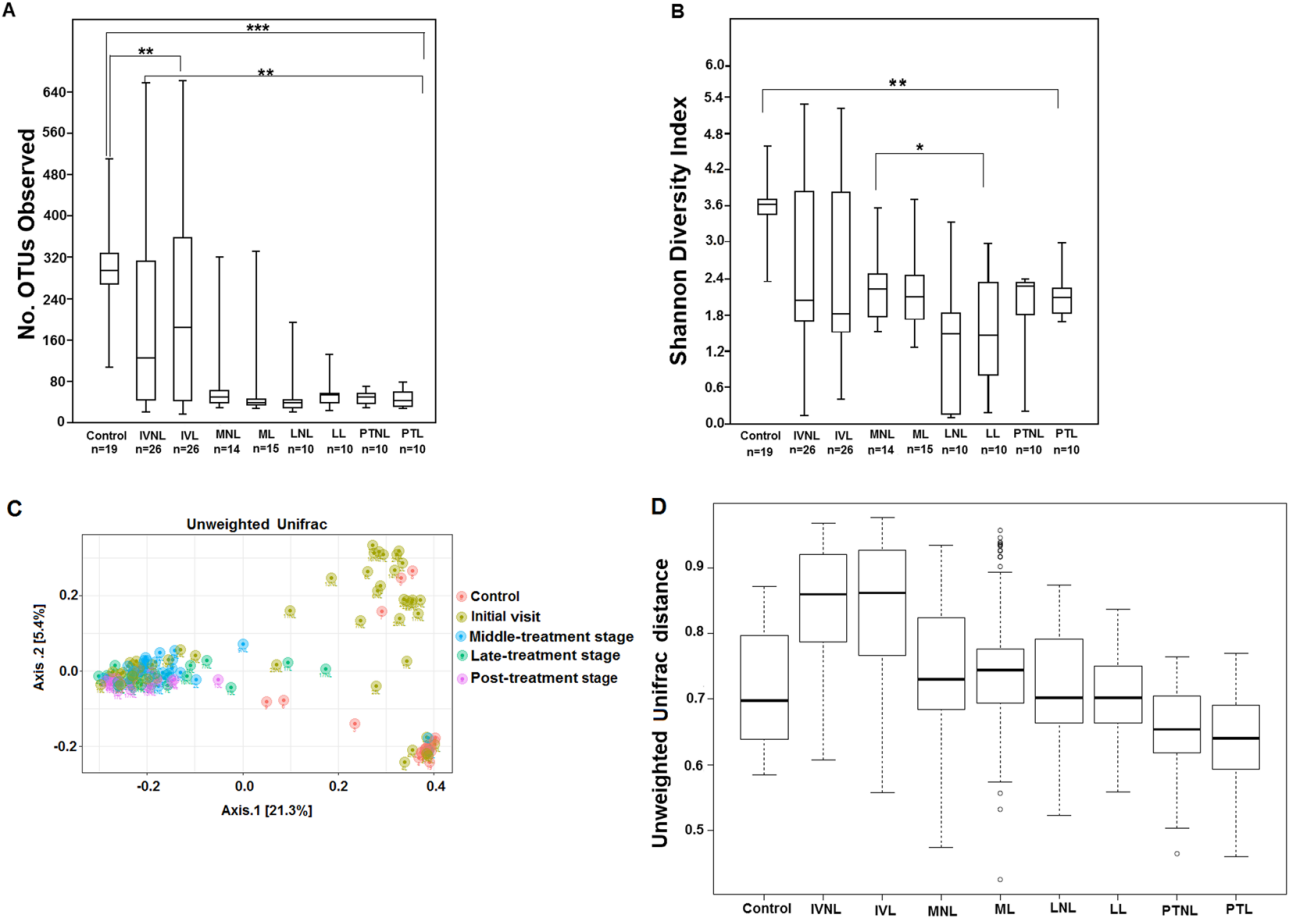
Alpha and beta diversity indices of the skin microbiome from control and patient groups. (**A**) A boxplot of the number of observed OTUs. (**B**) Boxplot based on Shannon diversity index. (**C**) Principal coordinate analysis of unweighted UniFrac, representing the distance between the skin bacterial communities from the control and patient groups. (**D**) Unweighted UniFrac distance boxplots showing differences between the control and patient groups and within patients longitudinally. For all the boxplots, the centerlines indicate the median, with the lower and upper edges of each box representing the first and third quartiles, respectively. The outliers are displayed as dots. Patient groups are named for the initial visit (IV), middle-treatment stage (M), late-treatment stage (L) and post-treatment (PT); and they are non-lesional (NL) and lesional (L). The P values were calculated by Wilcoxon rank sum test (*p < 0.05 **p < 0.01 and *** p < 0.0001), and adjusted by Bonferroni correction.

The alpha diversity was assessed by the number of observed OTUs and the Shannon diversity index. A significant difference (p < 0.05) was observed between the control and patient samples (Fig. 1A,B), with the former presenting the highest richness and diversity. The lowest OTU numbers were detected in patients across the MDT and PT stages, which was confirmed by the species accumulation curves (Fig. S1A,B). Pairwise comparisons between the IV, M, L and PT stages revealed significant changes in richness only between the IV and the next treatment stage samples (p < 0.01). Moreover, the Shannon diversity index showed a significant difference between the M and L stages (p < 0.05).

Species-accumulation curves showed that healthy skin harbors a significantly higher microbial diversity than leprous skin (q < 0.06; Fig. S1A). Moreover, the species-accumulation curves of the M, L, and PT stages reached a plateau and were close to saturation, with the PT stage exhibiting the lowest richness (Fig. S1B). These findings confirmed the alpha diversity results, as shown in Fig. 1A,B.

For the beta diversity analysis, an unweighted UniFrac distance matrix was calculated and ordinated by PCoA. Overall, the unweighted UniFrac-PCoA results (Fig. 1C) did not show a clear separation between the control and IV, which was highly diverse. By contrast, samples from M, L and PT formed a well-defined cluster (except in a few samples), presenting complete separation from the control. As illustrated in Fig. 1D, the pairwise unweighted UniFrac distances of the skin communities were significantly different between the control and IV samples (p < 0.05), and the distances progressively decreased throughout the treatment.

In summary, the alpha diversity analysis showed that the healthy skin microbiome is more diverse than that of leprous skin. Furthermore, in both the alpha and beta diversity analyses, significant differences were not observed between lesional and non-lesional skin for any investigated stage (p > 0.05).

### General features of the healthy skin microbiome

**Figure 2:**
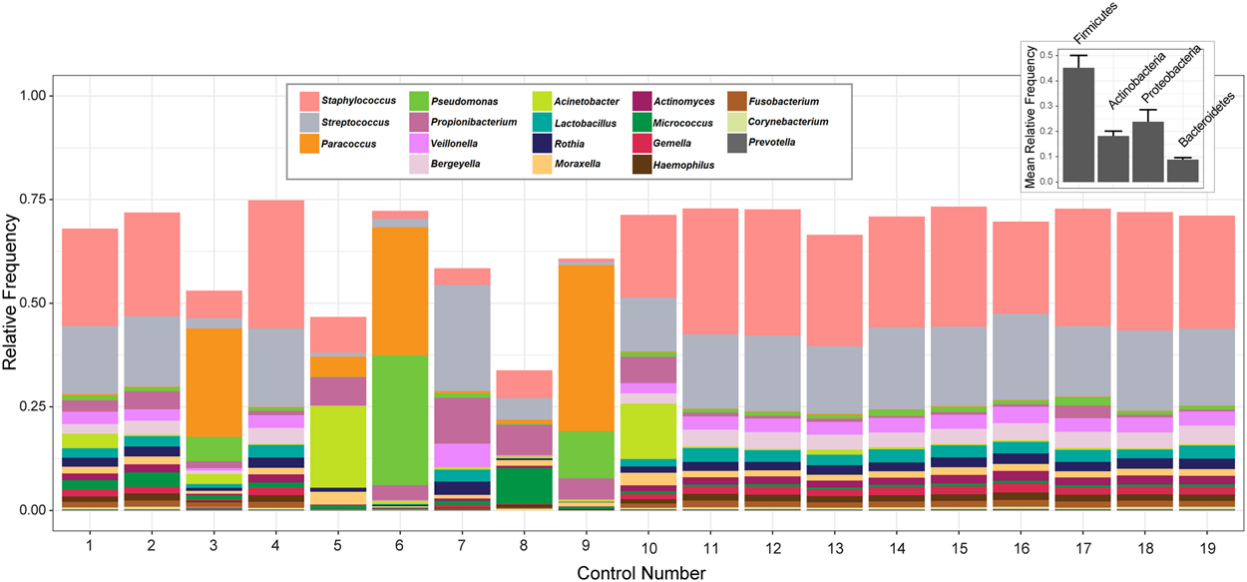
Skin bacterial community composition at the phyla and genera levels in control group. Data include the four major phyla and genera with a relative frequency ≥1%.

A phylum- and genus- levels taxonomic profile of the healthy skin microbiome is shown in Fig. 2. *Firmicutes* was the most abundant phylum in these samples, followed by Proteobacteria, Actinobacteria, and Bacteroidetes, together totaling 96.4% of all the reads.

Most OTUs (91.1% of all reads) were assigned to known genera (815), the abundance of which varied across individuals. Genera with a relative frequency >1% accounted for 74.6% of the community (Fig. 2). The dominant OTU was related to the *Staphylococcus* genus (24.2% of all the reads), which is considered a regular member of the human skin. Other common Firmicutes OTUs included the *Streptococcus* (16.6%), *Veillonella* (3%) *Lactobacillus* (2.5%) and *Gemella* (1.6%) genera.

Proteobacteria and Actinobacteria had similar frequencies (17.6% and 15.7%, respectively). *Paracoccus*, *Pseudomonas*, *Haemophilus* and *Moraxella* (Proteobacteria) ranged from 1–2.5% of all the reads. Within Actinobacteria, the skin-associated genera *Corynebacterium* (2.8%) and *Propionibacterium* (2.2%) prevailed, followed by *Rothia* (2.1%) and *Actinomyces* (1.7%). The most abundant genera of Bacteroidetes were *Bergeyella* (3.3%) and *Prevotella* (2.5%). Although the Fusobacteria phylum was detected at a low frequency (1.4%), it was represented almost exclusively by the *Fusobacterium* genus (1.3%).

The dominant OTUs of these genera were further classified at the species level using 16S rRNA gene sequences from the PATRIC database, and they included *Staphylococcus aureus*, *Streptococcus oralis, Pseudomonas stutzeri*, *Rothia dentocariosa* and *Prevotella melaninogenica* (Table S2).

### Differences between healthy and leprous skin microbiomes

**Figure 3:**
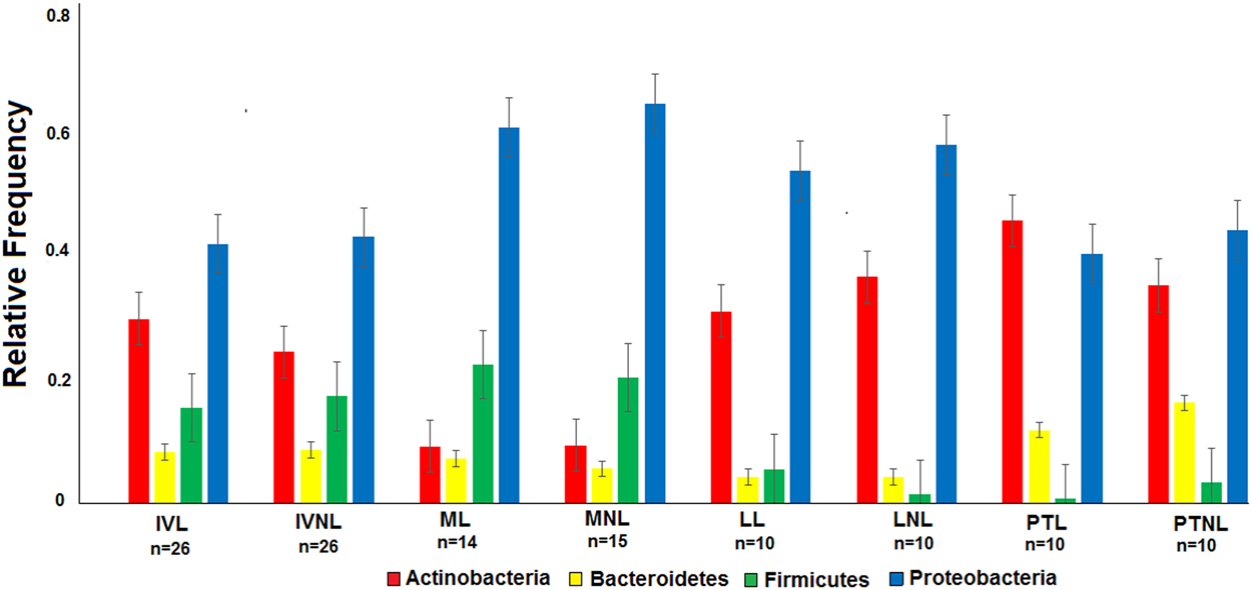
Skin bacterial community composition of leprosy patients who were sampled longitudinally. Data include the four major phyla.

The same four major phyla that inhabited healthy skin were observed in the IV patients, although their relative abundances varied significantly (Fig. 3). Proteobacteria (p < 0.05) was the most abundant phylum populating leprous skin, while Firmicutes, which was prevalent in healthy individuals, was underrepresented (p < 0.05). Actinobacteria was more abundant in leprous than in healthy skin. As observed in the control group, Bacteroidetes was also detected to a lesser extent (<10%). At the phylum level, the relative abundance was similar between lesional and non-lesional communities, confirming the alpha and beta diversity analyses.

**Figure 4:**
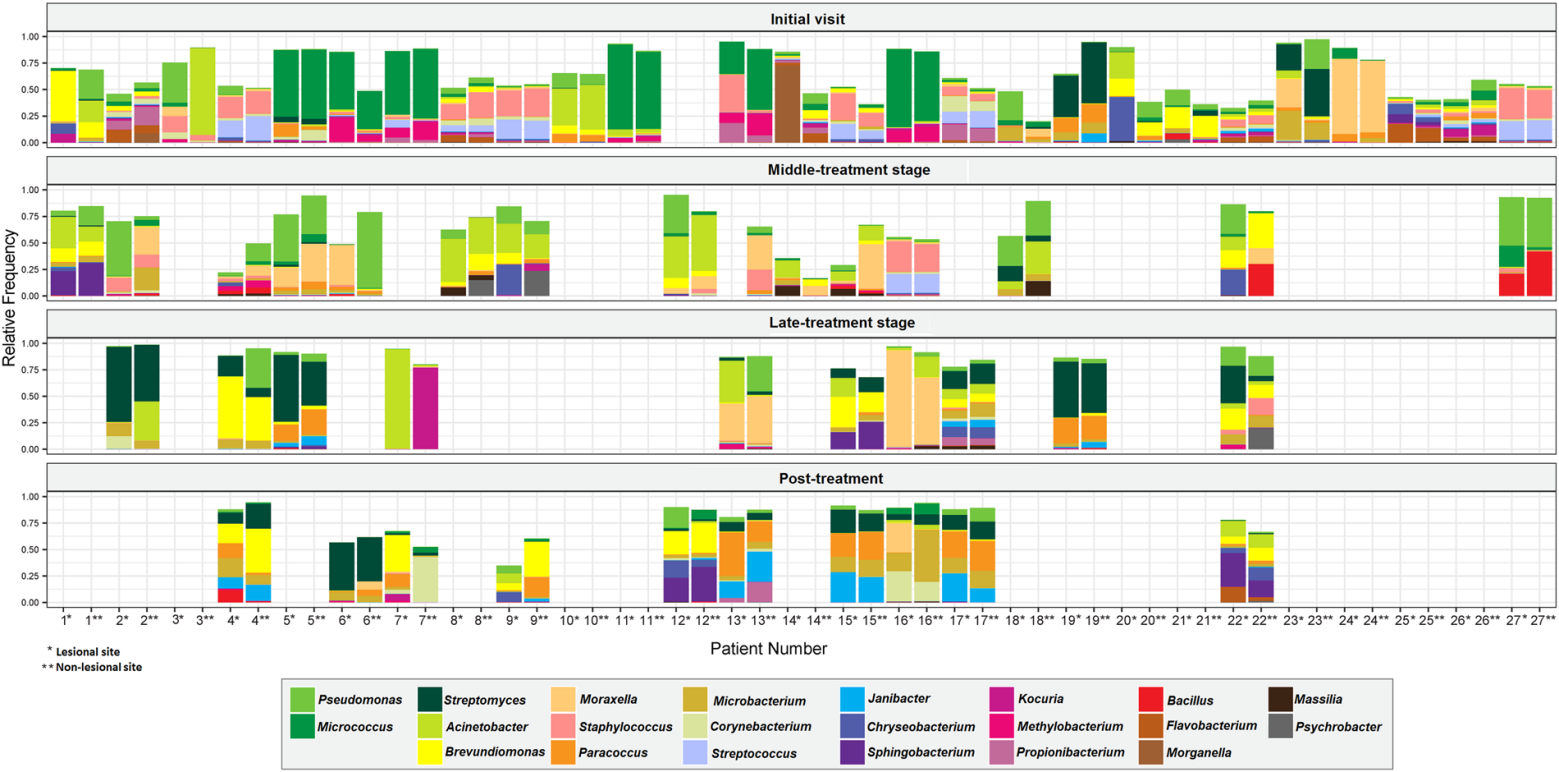
Skin bacterial community composition of leprosy patients sampled longitudinally. In total, 121 samples from the initial visit, therapy stages and post-treatment patients are represented by the 22 most frequent (>2%) genera in at least one community.

**Figure 5:**
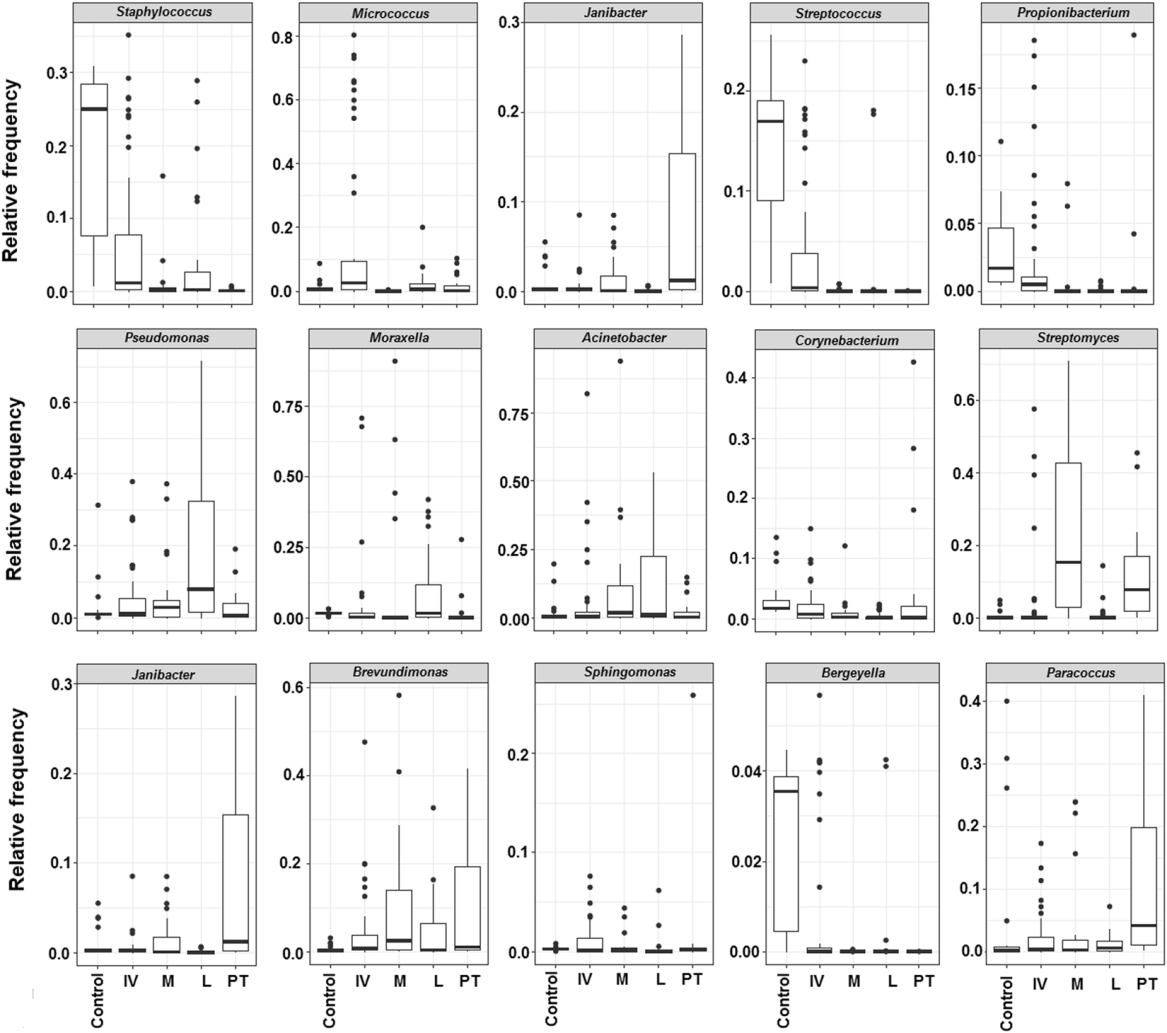
Selected genera and their distribution in >1% mean relative frequency among the control and patients. For all the boxplots, the centerlines indicate the median, with the lower and upper edges of each box representing the first and third quartiles, respectively. The outliers are displayed as dots.

The relative frequency at the genus level is shown in Figs 4 and 5. The leprous skin microbiome from IV patients was heterogeneous and strongly personalized. Although leprous skin presented many of the most frequently found genera in healthy skin, their proportion differed, as observed at the phylum level. For example, although healthy skin was dominated by one major genus (*Staphylococcus*), for most patients, there was no single dominant taxon. Moreover, some low-abundance genera (<0.3%), including the potential pathogens *Finegoldia*, *Neisseria*, *Porphyromonas* and *Ochrobactrum* and other uncommon skin taxa, were also highly personalized among IV patients.

Fifty-one OTUs with relative frequencies ≥0.5% were identified and classified in the control (34 OTUs assigned to 27 genera and 1 family) and IV (33 OTUs assigned to 25 genera and 2 families) groups. Overall, there was a variation in the relative frequency of the dominant OTUs (0.5 to 10.9%), some of which were absent or rare in the healthy and IV leprous skin bacterial communities. Among the 51 dominant OTUs, four were absent (*Lysobacter* OTU000060, *Moraxellaceae*, OUT000019 and *Chryseobacterium* OTU000049) or rare (*Morganella*, OTU000003 <0.03% of all the reads) in healthy individuals. Moreover, 15 out of the 51 that were observed in both the control and IV samples had relative frequencies ranging from 4- to 10-fold between these groups (e.g., *Staphylococcus*, 24.2% and 6.4%; and *Micrococcus*, 1.1% and 10.9%, respectively). Noticeably, OTUs with frequencies ≥0.5% from IV patients were represented by the *Morganella*, *Methylobacterium*, *Flavobacterium*, *Brevundimonas*, *Kocuria*, *Sphingomonas*, *Luteimonas*, *Streptomyces*, *Lysobacter*, *Chryseobacterium* and *Stenotrophomonas* genera. Some of them are potential pathogens (e.g., *Morganella morganii*, Table S2) and have been described in very different environments (e.g., *Luteimonas* is observed in aquatic habitats, soil or plant surfaces, and *Morganella* is found in the human intestinal tract). Notably, *Morganella* accounted for 7% of all the reads in IV patients.

**Figure 6:**
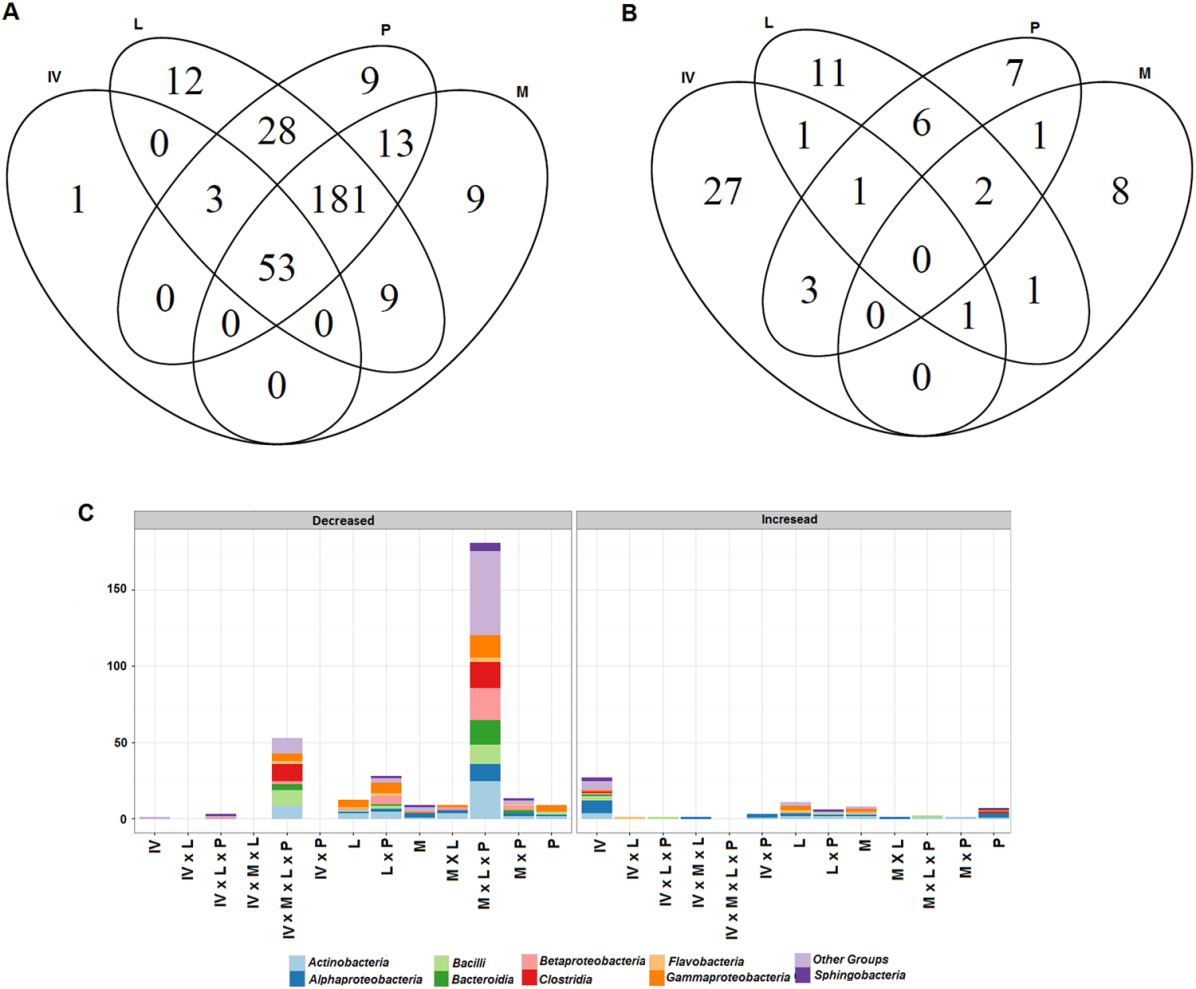
Venn diagram showing significantly decreased (**A**) and increased (**B**) OTUs of each patient group compared to the control, and their taxonomic assignment at the class-level (**C**), as given by Metastats. IV, initial visit; M, middle-treatment stage; L, late-treatment stage; and PT, post-treatment stage.

To identify the OTUs for which the frequencies varied statistically in the IV group compared to the control, a Metastats analysis was performed (Fig. 6). Overall, 90 OTUs showed significant differences, of which 57 decreased and 33 were enriched. Eight highly abundant OTUs (with a mean relative frequency ≥2%) were decreased (*Staphylococcus*, *Streptococcus*, *Veillonella*, *Prevotella* and *Bergeyella*) or enriched (*Micrococcus* and *Brevundimonas*) in the IV community.

Altogether, the results indicate that although healthy and leprous skin communities present many common taxa, there are exclusive features within each group, possibly as an effect of the perturbation triggered by the disease. Furthermore, many of the low-frequency genera observed in these patients are opportunistic pathogens. In this way, it is possible to conclude that the microbiomes of healthy and leprous skin have different characteristics.

### Multi-drug therapy impact on the composition and dynamics of the leprous skin microbiome

A shift in the distribution of the four major phyla was observed during the MDT in comparison to the initial visit. The Proteobacteria abundance (p < 0.05) increased significantly at the M- and L-treatment stages, returning to frequencies similar to that of the IV in PT (Fig. 3). By contrast, Firmicutes showed a dramatic decrease during the L and PT stages. The Actinobacteria abundance was lower at the M-treatment stage compared to the IV, and it increased from the L-treatment stage (p < 0.05), along with Bacteroidetes (p < 0.05). Significant differences were also found at the genus level. Although the patient’s skin microbiome was highly diverse at the IV, reduced interpersonal variation was observed during the MDT (Fig. 4). Increases in *Pseudomonas*, *Moraxella*, and *Acinetobacter* were observed in the M-treatment stage, followed by their decrease during the PT stage. At the L-treatment stage, a depletion in *Micrococcus* (0.3%) along with an increase in *Streptomyces* (23.9%) can be seen in Figs 4 and 5.

To identify how MDT affected specific taxa, pairwise comparisons between different groups were conducted with Metastats. As expected, the beginning of the therapy was followed by a significant decrease in many OTUs when compared to the control community (Fig. 6). Moreover, several low-abundance OTUs associated with the Clostridia, Alphaproteobacteria (*Methylobacterium*, *Bosea* and *Sphingomonas*), and Gammaproteobacteria (*Psychrobacter* and Pseudomonadaceae) classes were also significantly enriched throughout and after the treatment.

Among the 20 most abundant OTUs in the control (68.5% of all reads) and IV patients (57.9% of all reads), 12 OTUs (*Streptococcus*, *Veillonella*, *Gemella*, *Lactobacillus, Bergeyella*, *Prevotella, Rothia, Actinomyces, Haemophilus, Corynebacteriaceae, Fusobacterium*, and *Prevotellaceae*) and 6 OTUs (*Veillonella*, *Lactobacillus*, *Flavobacterium*, *Methylobacterium*, *Morganella* and *Moraxellaceae*) respectively, were nearly absent (>0.002%) in the PT, suggesting that MDT exerts a strong selective pressure on the indigenous skin microbiome. It is worth noting that the *Paracoccus* genus was the first taxon to thrive in the PT, reaching 10.3% of all the reads. Moreover, the predominant OTU in the L and PT samples was classified as *Streptomyces*. In summary, Firmicutes was the most MDT-impacted phylum, followed by Actinobacteria, Bacteroidetes and Proteobacteria.

## Discussion

Currently, studies on the skin microbiome are mostly from western countries with fewer from Asian countries, and the skin microbiome of different human populations remains largely unexplored. From a large 16S rRNA gene dataset, we provide the first description of the healthy skin microbiome and the dysbiosis signatures of leprosy in Brazilian individuals as well as the response of the skin microbiome to MDT. Moreover, the findings reported herein could contribute to the development of novel promicrobial and antimicrobial therapeutic approaches for treating leprosy.

Comparisons of microbiomes from healthy individuals and patients at different stages of MDT revealed a significant decrease in the alpha-diversity (in terms of the species richness and Shannon diversity index) in the latter, including those from PT. This could be a result of a disturbance in the community caused by *M. leprae* and/or MDT. Previous studies that examined the microbiome in skin disorders, such as psoriasis and atopic dermatitis, also reported a decrease in lesional and non-lesional skin bacterial diversity compared to the control^23–25^.

It is well known that skin samples from left and right symmetric sites generally exhibit lower intrapersonal than interpersonal variation^14^. Moreover, the healthy skin microbiome remains relatively stable over time^18^. In the present study, the alpha and beta diversity analyses revealed no significant intrapersonal alterations, suggesting that the infection caused by *M. leprae* could have systemic consequences, affecting the whole skin microbial community even in clinically non-affected skin. Moreover, our results demonstrated the dysbiosis of both lesional and non-lesional microbiomes from patients, suggesting that the skin microbiome can be altered even without symptom detection. Since the development of clinical leprosy may take decades^26^, the observation of shifts on the skin microbiome before the onset of symptoms has potential significance for early diagnosis. This prior diagnosis and therapy are the most important steps in preventing deformity and disability^26^.

Consistent with observations in previous studies on the healthy skin microbiome, Actinobacteria, Proteobacteria, Firmicutes and Bacteroidetes were the dominant phyla^2,17^ and were also observed in the dataset presented here. However, these studies reported a predominance of Actinobacteria, while Firmicutes (53.3%) prevailed in this study. Interestingly, Blaser *et al*.^27^ also reported an increased representation of Firmicutes members in the skin of Amerindians from Venezuelan Amazon. In this population, the dominant genus was *Staphylococcus*, which is in accordance with our study, whereas *Propionibacterium* was dominant in US residents. *Staphylococcus* has been shown to be dominant along with *Propionibacterium* in Chinese individuals^17^. By contrast, Clemente *et al*.^28^ studied the Yanomami Amerindian skin microbiome, and they did not find any single dominating taxon in spite of the high relative proportions of *Staphylococcus*, *Corynebacterium*, Neisseriaceae, and *Propionibacterium*.

The dominant OTU of healthy skin was classified as *S. aureus*, an opportunistic pathogen that is responsible for the majority of skin infections^29^. This species has been detected in healthy skin, although at a much lower proportion^17^ than that found here. By contrast, the enrichment of this species has been associated with atopic dermatitis^7^.

Other taxa that were observed to a minor extent or that were absent in healthy western individuals’ skin were detected at considerable proportions (1 to 16%) in this study, some of which are potentially pathogenic, namely *Streptococcus oralis* and *Moraxella osloensis* (endocarditis)^30,31^, *Prevotella bivia* (endometritis and perirectal abscesses)^32^ and *Bergeyella*. Importantly, this is the first report of *Bergeyella* inhabiting human healthy skin. *Janthinobacterium* was absent, in contrast to its appearance in American individuals as the dominant genus^12^. Altogether, these results support the idea that compositional differences can be attributed to factors such as ethnicity, lifestyle and/or geography, as highlighted in a review by Hannigan and Grice^33^.

Previously, the deep sequencing of 16 S rRNA genes has identified differences in the composition of cutaneous disorder microbiomes compared with healthy individuals^3^. Consistent with these data, leprous skin has a different microbiome than healthy individuals (Figs 3–5). The observed changes included a significant increase in Proteobacteria and an expressive reduction in Firmicutes in both lesional and non-lesional sites. Similar observations were made by Silva *et al*.^4^, who studied the microbiome of leprous lesions from DNA that was extracted from skin biopsies of lepromatous leprosy fixed in paraffin blocks. In fact, Grice *et al*.^12^ reported that the different sampling methods (i.e., swabs, scrapes, punches and biopsies) used to investigate the microbiome capture the same dominant phylotypes.

The skin is constantly exposed to environmental bacteria that can become transient and resident members of the host community, some of which are potentially pathogenic^14^. The inter- and intra-species interactions can shape and modulate the innate immune response of the host, which is essential for healthy skin^34^. Associations with a disease can be identified by the presence of specific taxa, as previously described for psoriasis and atopic dermatitis. The leprous skin microbiome was characterized by a significant depletion in the indigenous skin taxa, e.g., *Staphylococcus* and *Streptococcus*, and the enrichment of *Micrococcus* and *Brevundimonas*. These findings suggest an association of specific taxa with leprous skin. It should be noted that *M. leprae* was not detected, possibly due to the sampling procedure (skin swab), since this bacterium is an obligate intracellular pathogen of macrophages^35^.

Bacterial communities are perturbed by antibiotic use, after which they may return to their original composition (resilience, i.e., the capacity of a system to recover to its normal state after the perturbation has been removed) or functionally similar taxa (redundancy)^36^. Patients who were treated for 12 months with a combination of rifampicin, clofazimine and dapsone exhibited a shift in their microbiome that persisted for up to 5 months after the last sampling without MDT. Treatment-associated changes indicated a loss of diversity and a shift in the community composition, strongly impacting the relevant indigenous bacteria (Figs 1 and 5 and S1). These findings suggest a community with a slow recovery after MDT interruption. To assess the microbiome resilience, long-term monitoring would be needed. Studies showed that after antibiotic cessation, resilience in the human intestinal microbiome was observed, with the *Bacteroides* community not returning to its original composition for up to 2 years after treatment ended^37^.

It is well known that antibiotics affect not only the target pathogens but also resident taxa, selecting for resistant bacteria^38^. It is worth noting that the interpersonal variation was minor in patients from samples taken during and after the MDT compared to the IV. Furthermore, in the L stage, a significant increase in *Streptomyces* was observed, which could be explained by the fact that members of this genus are rifamycin producers that harbor resistance elements for self-protection^39,40^, which could favor their survival during MDT.

In conclusion, pronounced compositional differences in healthy skin microbiomes compared to other western and Asian populations were observed. These findings emphasize the importance of studying populations from distinct geographical and cultural settings, contributing to pan-microbiome comprehension. Major taxonomic changes and different abundance levels were evident in the leprous microbiome compared to the control. Molecular signatures associated with leprosy are potentially significant for early diagnosis. The MDT markedly reduced the diversity of the microbiome, with significant effects on indigenous taxa. This pervasive disturbance had prolonged effects, leading to a delay in the restoration of a healthy microbiome, which is of clinical relevance since indigenous taxa are essential to host health. Altogether, these results contribute to our knowledge of the global human skin microbiome and provide new insights into *M. leprae* effects on the microbiome. However, it should be kept in mind that this study represents a minor portion of the Brazilian population, which is characterized by high ethnic diversity. Finally, when accounting for the complexity of the leprous skin microbiome, future studies on a larger number of patients will be needed to strengthen and expand our conclusions.

## Methods

### Ethics statement

This study was approved by the Research Ethical Committees of the Universidade Federal de Minas Gerais (Belo Horizonte, Minas Gerais, Brazil) and the Hospital Eduardo de Menezes (reference number CAAE - 16784613. 9.0000.5149). After being informed about the study, all the participants gave their written consent between March 2014 and June 2016. The samples were anonymized for researcher use before analysis.

### Study design

A prospective study of multibacillary and paucibacillary leprosy patients who were clinically categorized using defined criteria^41^ was conducted to provide a thorough understanding of the leprous skin bacterial community composition and its shifts in response to MDT (rifampicin, clofazimine and dapsone). Sampling was performed at four time points: at an initial visit, the middle-treatment stage (between the 5^th^ and 9^th^ months of MDT), the late-treatment stage (between the 10^th^ and 12^th^ month of MDT) and post-treatment (between the 1^st^ and 5^th^ month following the end of the MDT). A total of 27 multibacillary (n = 26) and paucibacillary (n = 1) leprosy patients who were 30 to 75 years old from the Hospital Eduardo de Menezes (Belo Horizonte, Minas Gerais, Brazil) and similarly aged healthy volunteers (n = 19) were recruited. The inclusion criterion for patients was that each one had not received MDT prior to the study. The exclusion criteria for healthy controls included the use of systemic antibiotics in the preceding four months and prior chronic skin disorders, such as psoriasis or atopic dermatitis.

### Collection of the microbiome and DNA extraction

Swabs of lesional and non-lesional skin (symmetric dry sites, n = 27) from leprosy patients were taken during at least one stage of the investigation, according to the collection map of each patient (Table 1). For healthy individuals (n = 19), arm skin swabs were collected one time. The sampling procedure was performed with a sterilized brush that had been previously moistened in a sterile phosphate-buffered saline solution (PBS) in accordance with the Human Microbiome Project guidelines^42^. The samples were named according to the individual (by number, leprous or healthy volunteer, site (lesional, L and non-lesional, NL), and investigated stage (initial visit, IV; middle-treatment stage, M; late-treatment stage, L; and post-treatment, PT), in this order (e.g., 1LM, patient 1, lesional site, middle-treatment stage). The numbers of patients were 26, 15, 10 and 10 in the IV, M, L, and PT groups, respectively. The variation in the number of patients at the different investigated stages can be attributed to the abandonment of the MDT as well as to unexpected changes in a patient’s medical center attendance schedule, which were not previously mentioned to the researcher.

The swabs were placed directly in 300 μL of PBS and stored at −20 °C until DNA extraction. Prior to the extraction, the swabs were thawed and vortexed for 1 min. The resulting suspensions were centrifuged for 30 min at 10,000 g. The pellets were resuspended in 300 μL of PBS. The total DNA was extracted using a QIAamp® DNA Mini and Blood kit (QIAGEN, Germany) according to the manufacturer’s protocol. DNA quantification was performed with a Qubit® fluorometer (ThermoFisher Scientific, USA).

### 16S rRNA gene V3–V4 region amplification, library preparation and sequencing

To amplify the V3 and V4 hypervariable regions, the primers S-D-Bact-0341-b-S-17 (5′CCTACGGGNGGCWGCAG3′) and S-D-Bact-0785-a-A-21 (5′GACTACHVGG-GTATCTAATCC3′) were used, with Illumina adapter sequences^43^. The amplification reactions and library construction were performed according to the manufacturer’s instructions^44^. The paired-end sequencing of the libraries was performed on a MiSeq sequencer (Illumina, Inc., USA).

### Data processing

The raw sequence dataset was processed with MOTHUR v.1.33.0^45^ according to the MiSeq Standard Operating Procedure^46^. Reads with Q < 30, ambiguities, and homopolymers longer than 8 nucleotides were removed. Filtered reads were aligned and classified against the SILVA v.123 database^47^. Archaea, Eukaryota, mitochondrial, chloroplast, and unclassified reads were discarded. Chimeric reads were identified and removed using the UCHIME algorithm^48^. The remaining reads were grouped into operational taxonomic units (OTUs) by the average neighbor method, with 97% similarity. Singletons were removed from the dataset. A summary of this workflow is shown in Table S1. Additionally, to explore the presence of pathogenic bacteria, a BlastN comparison of dominant OTUs against 16 S rRNA gene sequences of known pathogens from the Pathosystems Resource Integration Center (PATRIC) database^49^ was performed. Only reads that showed 100% identity and alignment against a pathogen sequence were considered.

### Statistical analyses

Statistical analyses were performed using the R platform (https://www.r-project.org), with Phyloseq^50^. and Vegan^51^ packages. For alpha and beta diversity analyses, all the samples were rarefied to the lowest number of reads with the “rarefy_even_depth” Phyloseq command.

The similarity in OTU profiles among different communities was investigated through unweighted UniFrac distances and ordinated by principal coordinate analysis (PCoA). The non-parametric Wilcoxon rank-sum test was used to determine the statistically significant compositional and diversity differences between the control and patient groups, with p-values adjusted by Bonferroni correction for multiple testing. To detect the OTUs that statistically decreased or increased in each patient group compared to the control, the Metastats tool^52^ as implemented in MOTHUR was applied. For that purpose, samples containing less than 7,000 reads were discarded. The remaining reads were normalized with the “rarefy_even_depth” Phyloseq command, and the 500 more frequent OTUs were selected. Furthermore, the results were filtered for q-values < 0.06.

### Sequence accession

Sequencing reads are publicly available through the NCBI Sequence Read Archive (SRA; http://www.ncbi.nlm.nih.gov/sra) under accession number PRJNA390784.

### Data availability

The authors declare that the data generated in this study are available in the NCBI Sequence Read Archive (project accession number PRJNA390784).

## Electronic supplementary material

Supplementary Tables S1 and S2

Supplementary Figure S1.

Supplementary Dataset 1
