# Supplementary material for: Insights into the skin microbiome dynamics of leprosy patients during multi-drug therapy and in healthy individuals from Brazil
Source: Sci Rep. 2018 Jun 8;8:8783. doi: 10.1038/s41598-018-27074-0 (PMC5993821; doi:10.1038/s41598-018-27074-0)
Supplement: Supplementary file 1 — Supplementary Tables S1 and S2 [file 41598_2018_27074_MOESM1_ESM.pdf]

Insights into the skin microbiome dynamics of leprosy patients during multi-drug therapy and in healthy individuals from Brazil

Paulo E. S. Silva, Mariana P. Reis, Marcelo P. Ávila, Marcela F. Dias, Patrícia S. Costa, Maria L. S. Suhadolnik, Bárbara G. Kunzmann, Anderson O. Carmo, Evanguedes Kalapotakis, Edmar Chartone-Souza and Andréa M. A. Nascimento

Supplementary Information

Supplementary Table S1. Summary of the dataset quality control from the healthy and leprous skin libraries.

**Workflow**	
Raw reads	43,907,427
Resulting reads after a quality cutoff of ≥ 30	35,125,942
Contigs	23,285,870
Resulting reads after removal of homopolymers, ambiguous bases and length outside the range of ≤252 and ≥254bp	13,681,657
Resulting reads after aligning	13,681,657
Resulting reads after removal of chimera, mitochondria and chloroplast and those outside the Bacteria domain	10,403,990

Supplementary Table S2. More frequent OTUs from control and initial visit patient groups that were classified at the species level using 16S rRNA sequences from the PATRIC database.

OTU	Species	Frequency relative (%)	
		Control	Initial visit
000001	*Micrococcus luteus*	1.1	10.9
000002	*Staphylococcus aureus*	24.2	6.4
000003	*Morganella morganii*	0.03	7
000005	*Streptococcus oralis*	10	2.8
000008	*Propionibacterium acnes*	2.3	2.8
000010	*Pseudomonas stutzeri*	1.8	1.3
000011	*Acinetobacter johnsonii*	0.8	1
000012	*Moraxella osloensis*	1.8	2.8
000013	*Brevundimonas nasdae*	0.3	1.8
000014	*Kocuria atrinae*	0.2	1.4
000015	*Paracoccus denitrificans*	2.5	1.5
000020	*Microbacterium paraoxydans*	0.2	1
000023	*Streptococcus thermophilus*	5.3	1
000037	*Prevotella melaninogenica*	1.6	0.4
000045	*Actinomyces johnsonii*	1.4	0.3
000046	*Fusobacterium gonidiaformans*	1.1	0.3
000047	*Gemella sanguinis*	1.6	0.3
000085	*Rothia dentocariosa*	1.9	0.4
000097	*Haemophilus influenzae*	1.3	0.3
000104	*Lactobacillus crispatus*	1.4	0.3
