# Supplementary material for: Insights into the skin microbiome dynamics of leprosy patients during multi-drug therapy and in healthy individuals from Brazil
Source: Sci Rep. 2018 Jun 8;8:8783. doi: 10.1038/s41598-018-27074-0 (PMC5993821; doi:10.1038/s41598-018-27074-0)
Supplement: Supplementary file 2 — Supplementary Figure S1. [file 41598_2018_27074_MOESM2_ESM.pdf]

Supplementary Information

Insights into the skin microbiome dynamics of leprosy patients during multi-drug therapy and in healthy individuals from Brazil

Paulo E. S. Silva, Mariana P. Reis, Marcelo P. Ávila, Marcela F. Dias, Patrícia S. Costa, Maria L. S. Suhadolnik, Bárbara G. Kunzmann, Anderson O. Carmo, Evanguedes Kalapotakis, Edmar Chartone-Souza and Andréa M. A. Nascimento

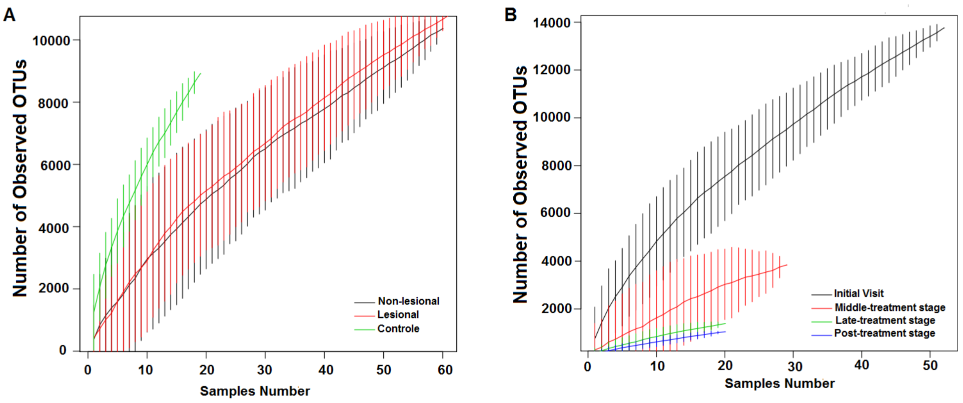

Supplementary Figure S1. Species-accumulation curves representing the number of OTUs (species) accumulated from the sample number for the control, and non-lesional and lesional samples **(A)** at the initial visit, therapy stages and post-treatment (**B**).
